# Alternative Management Approaches of Citrus Diseases Caused by *Penicillium digitatum* (Green Mold) and *Penicillium italicum* (Blue Mold)

**DOI:** 10.3389/fpls.2021.833328

**Published:** 2022-02-22

**Authors:** Usha K. Bhatta

**Affiliations:** Department of Plant Pathology, University of Georgia, Athens, GA, United States

**Keywords:** green mold, blue mold, citrus, post-harvest diseases, alternative control methods

## Abstract

Green mold (*Penicillium digitatum*) and blue mold (*Penicillium italicum*) are among the most economically impactful post-harvest diseases of citrus fruit worldwide. Post-harvest citrus diseases are largely controlled with synthetic fungicides such as pyrimethanil, imazalil, fludioxonil, and thiabendazole. Due to their toxic effects, prolonged and excessive application of these fungicides is gradually restricted in favor of safe and more eco-friendly alternatives. This review comprehensively describes alternative methods for the control of *P. digitatum* and *P. italicum*: (a) antagonistic micro-organisms, (b) plant extracts and essential oils, (c) biofungicides, (d) chitosan and chitosan-based citrus coatings, (e) heat treatments, (f) ionizing and non-ionizing irradiations, (g) food additives, and (h) synthetic elicitors. Integrating multiple approaches such as the application of biocontrol agents with food additives or heat treatments have overcome some drawbacks to single treatments. In addition, integrating treatment approaches could produce an additive or synergistic effect on controlling both molds for a satisfactory level of disease reduction in post-harvest citrus. Further research is warranted on plant resistance and fruit-pathogen interactions to develop safer strategies for the sustainable control of *P. digitatum* and *P. italicum* in citrus.

## Introduction

The citrus family Rutaceae primarily comprises sweet oranges, mandarins (tangerines), grapefruit, kumquats, lemons, limes, and pummelos. These important fruit crops are cultivated throughout the tropical and subtropical regions and hold an important economic position in the global fruit industry, with global production exceeding 98 million tons (United States Department of Agriculture, 2021). Oranges represent half of the production, followed by mandarins/tangerines, lemons/limes, and grapefruits. China, Brazil, the European Union, and the United States are the top citrus-producing countries (Liu et al., 2017; United States Department of Agriculture, 2021). Global citrus export in 2021 is estimated at 11 million tons, with oranges accounting for over 40% and mandarins/tangerines around 30%. South Africa is the largest exporter, followed by Turkey and Egypt, and the United States is the seventh-largest exporter (United States Department of Agriculture, 2021). The United States' share in global citrus trade is dropping primarily due to lower oranges exports. The citrus trade consists of two main markets: fresh and processed, in which oranges account for the most production. In addition to huge quantities of fruit juice, byproducts like essential oils, pectin, molasses, blend syrup, and dried pulp are essential components. Citrus flavonoids exhibit anti-cancer and anti-inflammatory properties and are widely used for medicinal purposes (Benavente-Garcia and Castillo, 2008; Talibi et al., 2014; Al-Snafi, 2016).

Several biotic and abiotic stressors in the post-harvest handling and citrus agroindustry such as picking, packaging, storage, transportation, and stocking have predisposed citrus fruits to mechanical wounds, resulting in the invasion of fruit-decaying microorganisms that cause spoilage, reduction in shelf life and value, and economic losses (Talibi et al., 2014). In general, the citrus fruit rot rate is around 10–30%, though it can increase to 50% in severe conditions (Ladaniya, 2011; Li et al., 2019; Youssef and Hussien, 2020). Losses from fungal decay of untreated fruits have been estimated as high as 90% during post-harvest handling and marketing (Smoot et al., 1971).

Of the many post-harvest diseases reported in citrus, two major challenges for the industry are green and blue mold (Kanetis et al., 2007; Talibi et al., 2014). Green mold, caused by *Penicillium digitatum* Sacc, and blue mold, caused by *Penicillium italicum* Wehmer, result in substantial economic losses around the globe (Kavanagh and Wood, 1971; Zamani et al., 2009). The *P. digitatum* and *P. italicum* fungi have a short disease cycle ranging from 3 to 5 days at 25°C and reproduce one to two billion conidial spores (Holmes and Eckert, 1999; Zhu H. et al., 2019).

*P. digitatum*, a necrotrophic fungus infecting citrus species *via* mechanical wounds and environmental factors such as cold, wind, insects, and hail, accounts for ~90% of total post-harvest losses alone (Perez et al., 2017; Zhu et al., 2017; Lin et al., 2019). The pathogen reproduces quickly on fruit surfaces, and its spores are ubiquitous in the atmosphere (Kanetis et al., 2007). The fungus penetrates fruit pericarp cells, spreads to the mesocarp, and invades nearby cells *via* germ tube (Han et al., 2013). The infected fruit then produces white mycelia and greenish conidia, a characteristic symptoms of green mold (Lin et al., 2019). The mycelium produces enzymes that break down fruit cell walls and initiate shrinkage, resulting in a sunken mummified form (Papoutsis et al., 2019). The infected pericarp and mesocarp cells plasmolyze, causing a soft watery spot, and the fruit is rotted (Han et al., 2013).

In the case of green mold, infection of the adjacent fruit is rare; however, spores may soil fruits. Ruptured oil glands in the wounded tissue emit volatiles (limonene, myrcene, alphapinene, and betapinene), organic acids, and sugar that stimulate conidial germination (Pelser and Eckert, 1977; Droby et al., 2008). *P. digitatum* produces thermogenic alkaloids, including tryptoquialanine A and tryptoquialanine C, which are harmful mycotoxins with potential risk to public health (Ariza et al., 2002; de Vilhena Araújo et al., 2019; Costa et al., 2021).

Spores of *P. italicum* are encapsulated in narrow white mycelium bounded by fluffy, water-soaked rind (Holmes and Eckert, 1999; Palou et al., 2002a,b, 2008; Talibi et al., 2014; Papoutsis et al., 2019). The white mycelium grows and digs into the infected tissue, sporulating blue conidia (Louw and Korsten, 2015). *P. italicum* is a nesting-type pathogen that spreads rapidly in packed containers to infect adjacent fruits (Ladaniya, 2008) even at lower temperatures in cold storage (Whiteside et al., 1993; Palou et al., 2002a; Iqbal et al., 2012, 2017) with reduced water availability (Plaza et al., 2003). Although initial lesions caused by green mold resemble blue mold disease, a thick non-sporulating mycelium limited by decaying peel surrounds green mold spores (Palou et al., 2008; Talibi et al., 2014).

**Figure 1:**
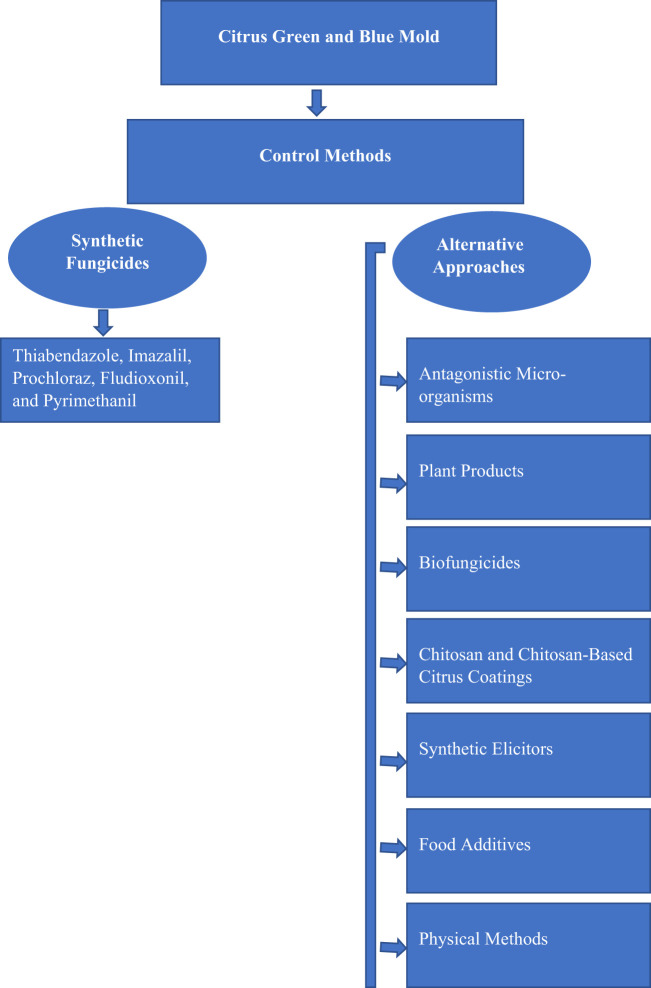
PRISMA flow diagram of the citrus green and blue mold management practices.

The economic losses caused by green and blue mold in citriculture are minimized using synthetic fungicides such as thiabendazole, imazalil, prochloraz, fludioxonil, and pyrimethanil, which are primarily used as control agents (Chen et al., 2019). The extensive use of synthetic fungicides has caused the proliferation of resistant strains of these phytopathogens and compromised the effectiveness of chemical treatments (Zhang X. et al., 2018; Chen et al., 2020a). Additionally, concerns about soil quality, environmental pollution, risks associated with human health, and accumulation of chemical residues in food have increased (Palou et al., 2008). Therefore, reducing harmful pressures on the environment is a key to creating a sustainable and healthy food system. Thus, an urgent search for safe and effective methods to replace and reduce the use of harmful chemicals to control *P. digitatum* and *P. italicum* is warranted. Alternative approaches and compounds from non-chemical sources are usually much less toxic to humans, safe, and eco-friendly when compared to chemical fungicides. These alternative methods include biological control with antagonistic microbes such as yeast, bacteria, and fungi, bio-fungicides, plant extracts and essential oils, chitosan, food additives and generally regarded as safe (GRAS) salts, synthetic elicitors, and physical methods such as heat or irradiation (Palou et al., 2016; Liu et al., 2017; Palou, 2018; Papoutsis et al., 2019). These approaches alone or combined with two or more methods represent a promising alternative to the existing chemical pesticides and offer a balanced solution for the control of citrus molds and sustainable production (Moraes Bazioli et al., 2019; Hulot and Hiller, 2021). The flow diagram for citrus green and blue mold management practices is shown in Figure 1.

Although several reviews on post-harvest diseases of citrus have been performed to date (Palou, 2009; Talibi et al., 2014; Chen et al., 2019; Moraes Bazioli et al., 2019; Papoutsis et al., 2019), no studies have reviewed all alternatives in a single paper. Further, the effectiveness and performance of these alternatives have not been discussed in detail. In order to fill this gap in the literature, this review paper aims to comprehensively review past and current research on alternative control methods and their successful application, implications for fruit quality, challenges, and future prospects. This review contributes to the literature on choosing appropriate alternative approaches to control post-harvest citrus disease. A wide range of scientific databases (Google Scholar, Scopus, PubMed, and SciELO) was utilized to search for articles on citrus post-harvest disease management. Based on the literature reviewed, a single treatment has been found less effective than two or more alternatives combined. I conclude that two or more alternatives can be combined to reduce disease incidence and extend shelf life of citrus fruits.

## Antagonistic Micro-Organisms

Biological control based on the application of antagonistic microorganisms has emerged as one of the most effective methods for controlling *P. digitatum* and *P. italicum* in citrus fruit. These microorganisms provide an effective eco-friendly choice to chemical fungicides and are easily accepted by consumers (Wilson and Eggemeier, 1991; Droby and Chalutez, 1999; Janisiewicz and Korsten, 2002; Droby et al., 2009). Microbial antagonists display protective and curative action comparable to synthetic fungicides. Antagonistic microorganisms such as yeast, bacteria, and some fungi have been widely used in the biological control of green and blue mold in citrus. An ideal microorganism should be genetically stable, effective at low concentrations, capable of surviving under adverse environmental conditions, inexpensive to formulate and produce, shelf-stable, resistant to common pesticides, compatible with commercial processing practices, and non-pathogenic to human health and the host (Wisniewski and Wilson, 1992). To suppress post-harvest pathogens, these microorganisms provide more than one control mechanisms, such as competition for nutrients and space (Droby et al., 1989, 2002; Panebianco et al., 2015), induction of resistance (Droby et al., 2002), secretion of specific enzymes and toxins and stimulation of secondary metabolism (Bar-Shimon et al., 2004; Luo et al., 2012), biofilm formation (Benhamou, 2004), antibiosis (Nunes et al., 2009), and mycoparasitism (Droby et al., 2002). Factors such as pH of growth media, type of strain used for the control, and time of application, such as pre- or post-harvest affect the efficacy of microbial antagonist (Droby et al., 2002).

Past studies have investigated the use of yeasts to control post-harvest fungal diseases (Platania et al., 2012; Kupper et al., 2013; Moretto et al., 2014; Ferraz et al., 2016; Liu et al., 2017). Yeasts have promising characteristics of pathogenic biocontrol because they infrequently produce antibiotic or mycotoxins substances that could leave residues on fruits (Droby et al., 2002; Gamagae et al., 2004; Zhang et al., 2005). Moreover, yeasts have been studied as antagonists for their inhibitory capacity to colonize surfaces for a long period. Some yeast strains such as *Wickerhamomyces anomalus, Saccharomyces cerevisiae, Rhodotorula minuta*, and *Aureobasidium pullulans* have been reported as “killer yeasts” with a killer phenotype: they produce killer proteins for suppressing pathogen development and deforming fungal hyphae (Comitini et al., 2009; Platania et al., 2012; Aloui et al., 2015; Ferraz et al., 2016). In mandarin oranges, the yeasts *W. anomalus, Metschnikowia pulcherrima*, and *A. pullulans* increase the activity of peroxidase and superoxide dismutase, thereby reducing the incidence and severity of *P. italicum* (Parafati et al., 2016). Other promising biocontrol agents against *P. digitatum* and *P. italicum* in citrus are bacteria such as *Bacillus subtilis* and *Streptomyces* sp. Bacterial pathogens such as *Bacillus* can act as antagonists or produce volatile organic compounds (VOCs) that increase plant resistance (Leelasuphakul et al., 2008). Maldonado et al. (2010) reported lemon fruit treated with *Streptomyces* RO3 metabolites showed fungicidal action and reduced the incidence of *P. digitatum*. Moreover, antifungal compounds such as 3-phenyllactic acid and benzeneacetic acid, 2-propenyl ester isolated from *Lactobacillus plantarum* IMAU10014 have exhibited antifungal activity against *P. digitatum* (Wang et al., 2012).

A bacterial strain SG-6 (identified as *Paenibacillus polymyxa*) was isolated as an endophyte from the root tissue of *Sophora tonkinensis*, which was highly efficient in reducing *P. digitatum* in citrus (Lai et al., 2012). Similarly, the application of endophytic *P. polymyxa* strain SG-6 in an *in-vitro* assay inhibited the growth of *P. digitatum* conidia and reduced decay in storage. This bacterium did not impair fruit quality parameters such as total soluble solids, ascorbic acid, titratable acidity, or firmness (Lai et al., 2012). In addition, a strain of *Bacillus amyloliquefaciens* HF-01, isolated from citrus fruit surfaces, was screened for *in vitro* antagonism toward *P. digitatum*. The isolate was further evaluated alone on artificially inoculated “Wuzishatangju” mandarin fruit. The isolate was found to perform significantly better than the water control in reducing the incidence of green and blue mold. Moreover, combination of tea saponin and *B. amyloliquefaciens* Hf-01 has been found to significantly improve the biocontrol activity of *B. amyloliquefaciens* HF-01. HF-01 combined with 50 μg mL^−1^ tea saponin was found to provide 90% control of green and blue mold without impairing any fruit quality parameters (Hao et al., 2011).

In a study by Wang et al. (2021b), citrus fruits with blue mold were treated with VOCs produced by *Pseudomonas fluorescens* ZX incubated on nutrient agar (NA) and in nutrient broth (NB). The study revealed that the VOCs from *P. fluorescens* ZX inhibited mycelial growth and conidial germination of *P. italicum* by 42.14% and 77.86%, respectively. Also, *in vivo* experiments showed that blue mold disease incidence and lesion size on citrus fruits were significantly suppressed by VOCs from *P. fluorescens* ZX incubated on NA, in NB, and on healthy fruits. Similarly, *in vitro* testing suggested organic acids and sulfur compounds were the active components of VOCs, with dimethyl disulfide and dimethyl trisulfide exhibiting the highest antifungal activity.

Studies have also examined the combined effect of multiple biocontrol agents: either different bacterial strains or bacteria with yeast/fungi, resulting in effective inhibition of citrus molds. For instance, the combined application of *Pseudomonas* and *Trichoderma* resulted in significant inhibition of *P. digitatum* on oranges and lemons (Panebianco et al., 2015). In addition, Meziane et al. (2006) investigated *Serratia plymuthica*, strains IC1270 and IC14 separately and in combination for inhibiting *P. digitatum* or *P. italicum* on oranges. A higher disease suppression and efficacy were observed when combined two bacterial strains (1 × 10^8^ cells/mL). Nutrient competition is considered the primary mode of action in strain IC1270, while antagonism requires a direct cell-to-cell interaction between IC14 and the pathogen.

Similarly, with the aim to inhibit mold and extend storage life in *Citrus reticulata* Blanco (“Xinyu” tangerine), one study immersed a biocontrol bacterium *Paenibacillus brasilensis* YS-1 into Xinyu tangerines. Xinyus soaked with *P. brasilensis* YS-1 for 10 min showed increased activity of peroxidase, superoxide dismutase (SOD), phenylalanine ammonia-lyase (PAL), and polyphenol oxidase (PPO) in comparison to the control water. This result shows that the post-harvest application of *P. brasilensis* YS-1 can control post-harvest decay, increase defensive enzyme activity, and maintain fruit quality (Chen et al., 2020c).

A few well-known fungal antagonists prove as effective as yeast and bacteria in controlling citrus mold diseases. Antagonistic fungi such as *Muscodor albus* and *Verticillium lecanii* produce volatile antimicrobial compounds that control *P. digitatum* decay (Benhamou, 2004; Mercier and Smilanick, 2005). Biofumigant *M. albus* has been used to fumigate lemons in storage to inhibit green mold (Mercier and Smilanick, 2005). Another study explored biocontrol activity of an entomopathogenic fungus *V. lecanii* on the pathogen *P. digitatum* at the cellular level (Benhamou, 2004). Treatment with *V. lecanii* in infected exocarp tissue had dramatic cellular changes characterized by rapid necrotization of the host exocarp cells with severely collapsed hyphae. This finding led to the hypothesis that molds exhibit fungus protection thanks to their direct antimicrobial properties and fruit-induced resistance (Benhamou and Brodeur, 2001). Some endophytic fungi produce volatile antifungal substances (Dennis and Webster, 1971; Strobel et al., 2001; Ezra et al., 2004). For instance, a fungus identified as *Nodulisporium* sp. CMU-UPE34 produced volatile antifungal compounds, namely alcohols, acids, esters, and monoterpene, with eucalyptol in the greatest abundance. *In vitro* tests showed the fungus killed a dozen different plant pathogens. *In vivo* mycofumigation with jasmine rice cultures of *Nodulisporium* spp. CMU-UPE34 was found to control decay by *P. digitatum* on *Citrus limon* and by *P. italicum* on *Citrus aurantifolia* (Suwannarach et al., 2013).

Combination with other treatment methods such as physical treatments, salts, and elicitors can boost the competency of biological control agents against citrus molds: *Candida membranifaciens* combined with ultraviolet irradiation and hot-water brushing (Terao et al., 2017); *Cryptococcus laurentii* with methyl jasmonate (MeJA) (Guo et al., 2014); *Kluyveromyces marxianus* with sodium bicarbonate (Geng et al., 2011); *Saccharomycopsis crataegensis* with sodium bicarbonate (Pimenta et al., 2010; see Table 1 for details). In recent work, Wang et al. (2021a) studied the joint application of *Meyerozyma guilliermondii* and an alginate oligosaccharide (bioactive compound from brown algae) as an effective method of controlling *P. italicum* on mandarin fruit. The study found that a combination of alginate oligosaccharide and *M. guilliermondii* provided better control than either treatment alone.

**Table 1:** Antagonistic microorganisms for the control of *P. digitatum* and *P. italicum*.

**Yeasts**	**Citrus species**	**Pathogens**	**Control mechanisms**	**References**
*Candida membranifaciens* combined with hot water brushing and ultraviolet irradiation	Oranges	*P. digitatum*	Induced systemic resistance on fruit peel. Provided additive effect, higher disease control efficacy, and extend fruit's shelf-life	Terao et al., 2017
Yeast isolates B13 and grape	Navel oranges, lemons and Valencia oranges	*P. digitatum*	Excellent control of *P. digitatum*, when applied to citrus fruit 48 h prior to artificial inoculation. Reduced decay of lemons and navel oranges, and <5% incidence on Valencia oranges, compared to >50% incidence in untreated fruits	Abraham et al., 2010
*Rhodotorula glutinis*	Oranges	*P. digitatum*	The higher efficacy of *R. glutinis* and increased competition for nutrients and space with pathogens	Zheng et al., 2005
*Cryptococcus laurentii* with methyl jasmonate (MeJA)	Mandarins *(Citrus reticulata* Blanco cv. Ponkan)	*P. digitatum*	*Cryptococcus laurentii* at 1 × 10^8^ cells/ml combined with 100 μmol/L, MeJA induced more natural resistance in fruit inoculated with *P. digitatum*. Greater activity of peroxidase, catalase, polyphenol oxidase, and rise in the mRNA expression level of PR5 (pathogenesis-related protein family 5) compared to the control	Guo et al., 2014
*Kluyveromyces marxianus* with sodium bicarbonate	*Citrus reticulata* Blanco cv. Wuzishatangju	*P. digitatum*	Compete with pathogens for nutrients and space. Salt stimulates *K. marxianus* growth and inhibits the germination of fungal spores	Geng et al., 2011
*Rhodosporidium paludigenum* (possess broad-spectrum antifungal effects) with ammonium molybdate	Satsuma mandarins	*P. digitatum*	Ammonium molybdate significantly increased the biological movement of *R. paludigenum* against green mold and reduced disease incidence by 89.3%. It reduced infection by suppressing proton-pump activity and spore germination of *P. digitatum*	Lu et al., 2018
*Pichia membranefaciens*	*Citrus sinensis* Osbeck cv. Jincheng	*P. digitatum* and *P. italicum*	Higher competition for nutrients and space with pathogen and induced host defenses	Luo et al., 2012
*Saccharomycopsis crataegensis* + sodium bicarbonate	Oranges	*P. digitatum*	Yeast and sodium bicarbonate alone lowered the disease severity by 41.7% and 19.8%, respectively. The combined applications impeded symptoms development from 2 to 10 days	Pimenta et al., 2010
*Pichia galeiformis* (BAF03)	Oranges, lemons	*P. digitatum*	Compete for space and nutrients Production of volatile organic compounds	Chen et al., 2020f, 2021
*Saccharomyces cerevisiae* ACBL-82 strain ACBL-76 strain, *Candida stellimalicola* ACBL-84 strain ACBL-87 strain *Meyerozyma caribbica* ACBL-86 strain	Orange fruits (*Citrus sinensis* cv. Lima)	*P. digitatum*	*S. cerevisiae* ACBL-82 strain, *C. stellimalicola* ACBL-84 strain, ACBL-87, and *S. cerevisiae* ACBL-76 inhibited more than 80% of pathogen mycelial growth *in vitro. M. caribbica* ACBL-86 strain, *S. cerevisiae* ACBL-82 strain decreased disease severity, and blocked green mold incidence in Lima sweet oranges	da Cunha et al., 2020
*Pichia membranaefaciens* combined with salicylic acid (SA)	*Citrus sinensis* L. Osbeck cv. Jincheng 447	*P. digitatum* and *P. italicum*	SA increased the yeast population in fruit wounds, facilitated *P. membranaefaciens* growth, and promoted nutrient and space competition. *P. membranaefaciens* in combination with SA effectively enhanced the synthesis of phenylalanine ammonia-lyase, chitinase, peroxidase, β-1,3-glucanase, polyphenoloxidase, and phenolics	Zhou et al., 2014; Spadaro and Droby, 2016
*Candida famata*	Oranges	*P. digitatum*	Induced resistance and stimulated the fruit to produce phytoalexins (scoparone and scopoletin). Enhanced rapid colonization of the fungal mycelium and the wounds	Arras, 1996
*Candida saitoana* with 0.2% glycolchitosan	Oranges, lemons	*P. digitatum*	Increased competition for nutrients and space. Glycolchitosan has antifungal and film-forming properties	El-Ghaouth et al., 2000
*Kloeckera apiculata*	Guoqing 1, Owari, Ponkan, and Newhall Navel Orange	*P. digitatum* and *P. italicum*	Competition for nutrients and space	Long et al., 2007
*Wickerhamomyces anomalus, Metschnikowia pulcherrima*, and *Aureobasidium pullulans* species with locust bean gum (LGB)	Mandarin fruit	*P. digitatum* and *P. italicum*	Competition for nutrients, antibiosis, fruit resistance induction, and killer activity. In combination with edible LGB coatings, yeast strains are effective for the long-term maintenance of biocontrol efficacy and yeast cell viability	Parafati et al., 2016
*Yarrowia lipolytica*	Mandarin oranges	*P. digitatum* and *P. italicum*	Induced higher activities of peroxidase, catalase, polyphenol oxidase, flavonoid, phenylalanine ammonia lyase compounds, and total phenols, which activates the defense mechanisms and improves resistance in mandarins	Zhu H. et al., 2019
*Candida oleophila*	“Marsh Seedless” grapefruit, *Citrus sinensis* (L.) Osbeck cv. Jincheng 447	*P. digitatum* and *P. italicum*	Nutrient competition, site exclusion, fungal cell wall degradation, resistance induction including accumulation of phytoalexins (scoparone, scopoletin, and umbelliferone), and direct mycoparasitism	Droby et al., 2002; Liu et al., 2019
*Metschnikowia citriensis*	*Citrus sinensis* (L.) Osbeck cv. Jincheng 447	*P. digitatum* and *P. italicum*	Deplete iron, adhesion, biofilm formation, and induce host resistance	Liu et al., 2019
*Saccharomyces cerevisiae* with calcium chloride (CaCl_2_)	Tarocco and Valencia oranges	*P. digitatum*	Competition for nutrients, space, and “killer” activity. Synergistic activity when applied along with CaCl_2_	Strano et al., 2002
*Cryptococcus laurentii* (1 × 10^7^ cells/mL) with cinnamic acid (1.5 mM)	“Orah” mandarins	*P. italicum*	Inhibits spore germination, loss of membrane integrity, and mycelial growth of *P. italicum*. Competition for nutrient and space	Li et al., 2019
*Pseudozyma antarctica*	*Citrus sinensis* (L.) Osbeck cv. Jincheng 447	*P. digitatum* and *P. italicum*	Fungal cell wall degradation, high lytic enzyme activity, and direct parasitism	Liu et al., 2019
*Candida stellimalicola*	Valencia sweet orange	*P. italicum*	The killer activity is a common biocontrol mechanism	da Cunha et al., 2018
*Debaryomyces hansenii*	Grapefruit, Mexican lime	*P. digitatum* and *P. italicum*	Competition for nutrients and space	Droby et al., 1989; Chalutz and Wilson, 1990; Hernández-Montiel et al., 2010
**Bacteria**				
*Bacillus amyloliquefaciens* HF-01 in combination with tea saponin	“Wuzishatangju” mandarin	*P. digitatum* and *P. italicum*	Colonization and secretion of antifungal peptides or other antibiotics or proteins by *B. amyloliquefaciens*. Plant growth promotion and systemic resistance	Kim and Chung, 2004; Wong et al., 2008; Hao et al., 2011
*Bacillus subtilis*	*Citrus reticulata* Blanco	*P. digitatum*	Antibiotics, proteins, secondary metabolites, enzymes, and volatile organic compounds have an inhibitory effect on mycelial growth and spore germination	Leelasuphakul et al., 2008
*Streptomyces* sp.	Oranges	*P. digitatum*	*In vitro* assays revealed competition for nutrients and space. Production of lytic enzymes acting on the fungus cell wall by altering growth and presence of antifungal metabolites	Najmeh et al., 2014
*Enterobacter cloacae*	“Gold Seal” orange (*Citrus sinensis* (L.) Osbeck)	*P. digitatum*	Competition for nutrients and space. The *E. cloacae* strain produces three volatile organic compounds: butyl acetate, 4,5-dimethyl-1-hexene, and phenylethyl alcohol, which inhibit conidial germination, and hyphal elongation	Chen et al., 2016
*Pseudomonas cepacia, Pseudomonas fluorescens, Pseudomonas corrugata*, and *Pseudomonas syringae*	Lemon fruit *(Citrus limon)*	*P. digitatum*	Antibiosis and competition for nutrients and space	Smilanick and Denis-Arrue, 1992
*Pantoea agglomerans* in combination with sodium bicarbonate	Oranges	*P. digitatum* and *P. italicum*	The exact mechanism of control is not clear. Colonization and parasitizing the pathogen through nutrient competition could be possible mechanisms	Riggle and Klos, 1972; Teixidó et al., 2001
**Fungi**				
*Muscodor albus*	Lemons	*P. digitatum*	Production of volatile compounds	Mercier and Smilanick, 2005
*Verticillium lecanii*	Lemons	*P. digitatum*	Fruit-induced resistance and antimicrobial activity	Benhamou, 2004

Citrus fruits can be infected either prior to harvest or during harvesting and processing; however, most studies on biocontrol agents have examined the post-harvest period, and only a few have studied the pre-harvest period. More work is needed on the application of these biocontrol agents before fruit harvest, as the timing of application impacts the effectiveness of the biocontrol agent. Application time is crucial as the biocontrol agent can utilize available nutrients otherwise consumed by the pathogen in the pre-harvest period (Luo et al., 2012; Panebianco et al., 2015; Papoutsis et al., 2019). So far, most studies have examined the antifungal activities of microbial antagonists as a stand-alone product. Results have shown inconsistent performance and control of previously established infections compared to many commercial fungicides (Ippolito and Nigro, 2000; Zheng et al., 2005). Integrated approaches combining biological control with other methods such as salts or food additives, physical treatments, and non-chemical elicitors or plant growth regulators are one of the most promising means of disease management (Huang et al., 1995; Droby et al., 1998; El-Ghaouth et al., 2000; Arras et al., 2002; Janisiewicz and Korsten, 2002; Porat et al., 2002; Zhang et al., 2004; Papoutsis et al., 2019). Overall, an integrated approach facilitates additive, synergistic, complementary, preventive, curative effects on citrus molds while minimizing fungicidal residues (Palou et al., 2008; Smilanick et al., 2008).

## Natural Plant Products

In recent years, plant extracts, essential oils, and natural compounds have been evaluated as an alternative chemical means for controlling citrus post-harvest decay (Table 2). More than 1,340 plant species are documented sources of antimicrobial compounds and novel botanical fungicides (Cowan, 1999; Tripathi and Dubey, 2004). Plant extracts are biodegradable, non-phytotoxic, generally safe for human health and the environment, inexpensive, and equally effective as chemical fungicides. Some phytochemicals of plant origin have been successfully formulated as botanical pesticides in integrated pest management programs (Tripathi et al., 2004). Plant extracts and essential oils obtained from medicinal and aromatic plants have effectively controlled agar plates (Ameziane et al., 2007) and wounded citrus fruits (Wilson et al., 1997; Mari and Guizzardi, 1998; Talibi et al., 2012). Plants contain secondary compounds such as acetaldehyde, ethanol, ethyl formate, ethyl benzoate, benzaldehyde, methyl salicylate, eugenol, jasmonates, glucosinolates, hexanal, thymol, allicin, isothiocyanates, citral, limonene, and a variety of phenolic compounds (for example, flavanones, polymethoxylated flavones, and coumarins) that possess antifungal property. These compounds have been derived from plants such as mint, cinnamon, thyme, clove, garlic, oregano, pomegranate, *Acacia* sp., *Aloe* sp., and citrus fruits (Utama et al., 2002; Tripathi and Dubey, 2004; Palou et al., 2008).

**Table 2:** Plant extracts, essential oils, and natural compounds for the control *P. digitatum* and *P. italicum*.

**Pathogens**	**Plants**	**Plant extracts**	**References**
*P. digitatum*	*Citrus reticulata* (mandarin)	Waxy components, hexane extract, tangeritin, citral, and nobiletin	Johann et al., 2007
	*Glycine max*	β-conglycinin and glycinin	Villalobos et al., 2016
	*Citrus japonica* (kumquat)	Scoparone and scopoletin	Rodov et al., 1992
	*Rosmarinus officinalis* L. (rosemary)	Essential oils, flavonoids, polyphenols, and methanol extracts	Hendel et al., 2016
	*Aloe vera*	Aloe saponins and anthraquinones	Sitara et al., 2011
	*Saliva fruticosa* Mill.	Carnosic acid, carnosol, and hispidulin	Exarchou et al., 2015
	*Coriandrum sativum, Cuminum cyminum, Cinnamomum zeylanicum, Thymbra spicata, Anethum graveolens* (dill), *Syzygium aromaticum, Cymbopogon citratus, Pelargonium graveolens, Mentha spicata* (spearmint), *Mentha piperita* (peppermint), *Foeniculum vulgare, Artemisia annua, Lavandula stoechas, Origanum syriacum, Lippia scaberrima*, and *Laurus nobilis* (laurel)	Volatile oil	Yigit et al., 2000; Hall and Fernandez, 2004; Soylu et al., 2005; Du Plooy et al., 2009; De Corato et al., 2010; Tyagi and Malik, 2011
	*Origanum dictamnus, Origanum vulgare, Origanum majorana*, and *Eugenia caryophyllata*	Essential oils, thymol, carvacrol, and eugenol	Daferera et al., 2000; Yahyazadeh et al., 2008
	*Parastrephia lepidophylla, Parastrephia phyliciformis*, and *Chuquiraga atacamensis*	Aqueous extracts and total phenolics	Sayago et al., 2012
	*Solanum nigrum, Sisymbrium irio, Ranunculus asiaticus, Crepis aspera, Chenopodium murale*, and *Ceratonia siliqua*	Aqueous extracts, and flavonoids alkaloids	Qasem and Abu-Blan, 1995; Qasem, 1996; Ameziane et al., 2007; Askarne et al., 2012; Musto et al., 2014
	*Astilbe myriantha* Diels, *Breonadia salicina*	Triterpenoid	Eloff and Mahlo, 2011; Song et al., 2011
	*Withania somnifera*	Caffeic acid, salicylic acid, and 3,4-dihydroxybenzoic acid	Mekbib et al., 2007, 2009
	*Acacia seyal*	Methanol extracts and gallic acid	Mekbib et al., 2007
	*Lippia rehmannii*	Verbascoside	Shikanga et al., 2009
	*Lippia graveolens*	Ethanolic and hexanic extracts	De Rodríguez et al., 2011
	*Sapium baccatum*	Tannic acid	Zhu H. et al., 2019
*P. italicum*	*Citrus aurantium* (sour orange)	Essential oils, α-terpineol, terpinen-4-ol, linalool, and limonene	Trabelsi et al., 2016
	*Ageratum conyzoides*	Essential oils	Dixit et al., 1995
	*Citrus sinensis* (sweet orange), *Citrus clementina* (clementine), and *Mentha arvensis* (wild mint)	Volatile oil, citronellal limonene, linalool	Del Río et al., 1998; Tripathi et al., 2004; Droby et al., 2008
	*Arenaria rubra* and *Acacia nilotica*	Aqueous extracts	Tripathi et al., 2002; Ameziane et al., 2007; Askarne et al., 2012
	*Ocimum canum* and *Zingiber officinale*	Volatile oil	Tripathi et al., 2004
	*Anvillea radiata, Asteriscus graveolens, Bubonium odorum*, and *Inula viscosa*	Petroleum ether, chloroform, and ethyl acetate extracts	Ameziane et al., 2007; Askarne et al., 2012, 2013
	*Halimium umbellatum*	Methanol extract	Ameziane et al., 2007; Askarne et al., 2012, 2013
	*Lantana camara*	Verbascoside	Oyourou et al., 2013
	*Populus* × *euramericana* cv. “*Neva”* (poplar buds)	Flavonoids of chrysin, pinocembrin, and galangin	Yang et al., 2016
	*Ficus hirta* Vahl's	Pinocembrin-7-o-beta-D-glucoside	Wan et al., 2017a
	*Simmondsia chinensis*	Oil emulsion	Ahmed et al., 2007
*P. digitatum* and *P. italicum*	*Citrus paradisi* (grapefruit)	Coumarins including scoparone, umbelliferone, osthol, seselin, auraptene 7-geranoxycoumarin; essential oils, citral, myrcene, α-terpineol, linalool, α-pinene, limonene, sabinene, and nootkatone	Afek et al., 1999; Droby et al., 2008
	*Citrus limon* (Lemon)	Waxy components, scoparone, hexane extract, and xanthyletin; limettin, 5-geranoxy-7-methoxycoumarin, isopimpinellin, and scoparone; citral, and volatile oil	Rodov et al., 1995; Johann et al., 2007
	*Aloe ferox, Aloe saponaria*, and *Aloe mitriformis*	Aloin	Zapata et al., 2013
	*Camellia sinensis*	Tea saponins	Hao et al., 2010
	*Plantago lanceolata* and *Sanguisorba minor*	Caffeic acid derivatives, flavonoids, and (iso) verbascoside	Gatto et al., 2011
	*Ramulus cinnamomi*	Cinnamaldehyde and cinnamic acid	Wan et al., 2017b
	*Allium sativum* (garlic)	Aqueous extracts, ethanolic extracts, allicin	Obagwu and Korsten, 2003
	*Melaleuca alternifolia, Zataria multiflora*, and *Thymus vulgaris* (thyme)	Essential oils	Ramezani et al., 2009; Fatemi et al., 2012; Zhang X. et al., 2018
	*Thymus capitatus, Thymus leptobotrys*, and *Thymus riatarum*	Volatile oil, carvacrol, and thymol	Arras and Usai, 2001; Boubaker et al., 2016
	*Cistus villosus*	Aqueous extracts	Ameziane et al., 2007; Askarne et al., 2012
	*Punica granatum*	Ethanol, methanol and water extracts, and phenolic compounds	Nicosia et al., 2016
	*Lippia javanica*	Verbascoside	Shikanga et al., 2009
	*Thymus broussonnetii* subsp. *hannonis*	Camphor and α-terpineol	Boubaker et al., 2016
	*Thymus satureioides* subsp. *pseudomastichina*	Borneol and thymol	Boubaker et al., 2016
	*Peganum harmala*	Harmine, tetrahydroharmine, and harmaline	Kanan and Al-Najar, 2009
	*Chinese propolis*	Ethyl acetate extract, methanol, and pinocembrin	Yang et al., 2010 Peng et al., 2012
	*Sonchus oleraceus, Borago officinalis*, and *Sanguisorba minor*	Methanol extract	Gatto et al., 2011

Plant extracts are known for their preservative, antimicrobial, and antifungal properties. Several studies have investigated aqueous plant extracts and organic solvent extracts against citrus post-harvest pathogens. Plant extracts from species *Withania somnifera* and *Acacia seyal* have resulted in the inhibition of *P. digitatum* in citrus by up to 70% under storage conditions (Samson, 1984). Similarly, flavonone pinocembroside compounds obtained from the fruit of *Ficus hirta* Vahl. have shown antifungal activity against *P. italicum* in “Navel” oranges by direct inhibition of mycelial growth *via* membrane targeting (Chen et al., 2020d). In addition, secondary compounds such as acetaldehyde, benzaldehyde, cinnamaldehyde, ethanol, benzyl alcohol, nerolidol, and 2-nonanone have shown effectiveness against *P. digitatum* (Utama et al., 2002). Citral, an active compound produced in the flavedo of citrus induced a strong defense mechanism against *P. digitatum* inhibiting mycelial growth and spore germination (Rodov et al., 1995; Klieber et al., 2002; Fisher and Phillips, 2008). Methanol extracts from *Sanguisorba minor* and *Cistus villosus* showed satisfactory control of *P. digitatum* (Ameziane et al., 2007; Gatto et al., 2011).

Likewise, *in vitro* and *in vivo* assays have demonstrated the capacity of 7-geranoxy coumarin, a natural compound of grapefruit, to act against *P. digitatum* and *P. italicum* (Agnioni et al., 1998). A recent study examined the antifungal potential of pinocembrin-7-glucoside (P7G) isolated from *Ficus hirta* Vahl. against *P. italicum*. The *in vivo* test showed P7G significantly inhibited mycelial growth in artificially inoculated “Newhall Navel” oranges. Further, P7G triggered a marked decline in both chitin and glucanase contents of *P. italicum* mycelia and destroyed the cell wall structure (Chen et al., 2020b). Ethyl extracts such as Chinese propolis ethyl acetate extract (PEAE) have been assessed to control *P. digitatum* and *P. italicum* on post-harvest citrus fruits. Studies show that PEAE strongly inhibited spore germination and mycelium growth, induced unusual morphological alterations, and reduced decay caused by *P. digitatum* and *P. italicum* on “Zhongqiu” mandarins (Yang et al., 2010). Recently, Zhu H. et al. (2019) investigated the antifungal activity of tannins, a natural polyphenolic compound on *P. digitatum*. *In vivo* tests showed a significant reduction of *P. digitatum* symptoms in artificially inoculated citrus fruit in storage conditions. The study demonstrated that the antifungal mechanism of tannic acid resulted in the disruption of the cell walls and the plasmatic membrane, causing leakage of intracellular contents such as sugars.

The role of plant extracts in combination with alternative treatments such as wax, oil, biocontrol agents, and thermotherapy has been examined against *P. digitatum* and *P. italicum*. For instance, Obagwu and Korsten (2003) tested the water and ethanol extracts of garlic clove alone or in combination with sunflower oil or fruit wax (Obagwu and Korsten, 2003). The authors found all treatment combinations effective; however, a 1% extract in sunflower oil being the most effective against *P. digitatum* and *P. italicum* in “Valencia” oranges and as effective as fungicides. Sukorini et al. (2013) investigated biofungicidal yeast *Candida utilis* TISTR 5001 combined with *Eugenia caryophyllata* crude extract to control *P. digitatum* in tangerines and suggested the application of these extracts as a potential disease management approach. In addition, Koltz et al. (2020) studied the effects of canola and mustard extracts and sachets alone and in combination with thermotherapy to control *P. digitatum*. The study found that the fungitoxic volatile compounds produced with canola and mustard extracts and sachets significantly reduced *P. digitatum in vitro* and in inoculated oranges.

Use of essential oils to control citrus green and blue mold is gaining importance, thanks to antimicrobial and antioxidant properties, synergistic effects, presence of active compounds, and low residue levels (Tripathi and Dubey, 2004; Bakkali et al., 2008). In a study by Chen et al. (2019), the efficacy of clove essential oil (CEO) against *P. italicum* was investigated. Application of CEO was found to inhibit *P. italicum* growth when used at 0.05%–0.8% (v/v). In addition to its direct antifungal activity, the CEO has demonstrably enhanced the activity of defense-related enzymes such as chitinase and peroxidase to induce host defense responses. Similarly, the influence of numerous essential oils such as lemon grass, eucalyptus, clove, and neem was investigated on “Kinnow” mandarin inoculated with *P. digitatum* and *P. italicum*. The results indicated that these essential oils inhibited both pathogens' growth/colony diameter over untreated PDA plates and reduced decay loss in storage; however, lemon grass oil produced the most potent effect (Jhalegar et al., 2015). Among essential oils, p-anisaldehyde is a naturally occurring and fragrant phenolic compound primarily isolated from anise, cumin, fennel, and garlic. A study was conducted to investigate the antifungal efficiency of p-anisaldehyde on the mycelial growth of *P. digitatum* and *P. italicum* on “Satsuma” mandarin. P-anisaldehyde exhibited a robust inhibitory effect on *P. digitatum* and *P. italicum* leading to altered mycelia morphology, cell wall integrity, and membrane permeability, with minimum inhibitory and fungicidal concentrations of 2.00 μl/ml (Che et al., 2020).

Applying essential oil-amended coatings to citrus is one of several approaches for the control of post-harvest pathogens while maintaining fruit quality. Commercial coatings modified with *Lippia scaberrima* essential oil achieved 100% efficacy against *P. digitatum* in “Tomango” oranges (Du Plooy et al., 2009). Yahyazadeh et al. (2009) stated that applying thyme or clove oil to the surface of oranges in polyethylene films lowered *P. digitatum* in vapor-phase experiments at 25°C. In other research, supplementing carnauba wax with *Cinnamomum zeylanicum* essential oil (0.5%, v/v) achieved 90% disease control against post-harvest *P. digitatum* and *P. italicum* in citrus (Kouassi et al., 2012). In addition, citral, a naturally occurring isoprenoid compound with two isomers (geranial and neral), provided antifungal activity against *P. digitatum* in “Navel” oranges (Wolken et al., 2002; Wuryatmo et al., 2003; Droby et al., 2008). Fan et al. (2014) found that citral combined with wax *in vitro* and *in vivo* decreased the incidence of *P. digitatum* in “Ponkan” mandarins with significant increases in Vc content and antioxidant enzymes (catalase, SOD, and peroxidase).

Generally, plant products provide antifungal activity and trigger defense mechanisms resulting in cell wall synthesis and elevation of the total soluble phenolic compound concentration of orange peels. These products serve as physical and biological barriers to invading pathogens by altering their membrane functionality (Lanciotti et al., 2004; Mekbib et al., 2007). However, the use of natural plant products to inhibit *P. digitatum* and *P. italicum* is in its initial phase as not much is known about their mechanisms of action against post-harvest pathogens. In addition, important limitations with regard to essential oils include potential phytotoxicity, low stability, and induction of strong odors or flavors in treated fruits (Palou et al., 2008; Talibi et al., 2014). More investigations into the mechanisms of such plant products are warranted to control citrus post-harvest diseases.

## Biofungicides

Biofungicides are formulations of biocontrol microorganisms used against plant diseases (Roger and Keinath, 2010). These fungicides are manufactured in different forms such as wettable powder, emulsion concentrate, suspension concentrate, and tablets, usually for soil drench and application on leaves, seeds, and roots. Biofungicides possess advantages such as low toxicity, low concentrations of active substances, enhanced plant resistance, effective colonization of wounds, nutrient competition, and cost-effectiveness. In 2020, the biofungicides global market was valued at $1.6 billion and estimated to grow at a compound annual growth rate of 16.1% to $3.4 billion by 2025 (Matrose et al., 2021).

Some biofungicides used in agricultural production are Binab-T (*Trichoderma harzianum* and *Trichoderma polysporum*), Serenada, Baktofit, Kodiak, Rhizo-plus, and Phytosporin (*Bacillus subtilis*), Phytolavin (*Streptomyces griseus*), and Planriz (*Pseudomonas fluorescens*) (Khakimov et al., 2020). Commercial biofungicides based on antagonistic yeasts available on the market to control *P. digitatum* and *P. italicum* include “Shemer” (*Metschnikowia fructicola*) (Kurtzman and Droby, 2001), “Aspire” (*Candida oleophila*) (Liu et al., 2013), and “Pantovital” (*Pantoea agglomerans*) (Vinas et al., 1998). Likewise, two biofungicidal products based on antagonistic bacteria are “Biosave” (*Pseudomonas syringae*) (Fravel and Larkin, 1996) and “Natamycin” (*Streptomyces* sp.) (Chen et al., 2020e). The biofungicide “Shemer” is reported to be as effective against molds in oranges as the chemical fungicide imazalil (Wisniewski et al., 2009; Piombo et al., 2018). The biofungicide “Aspire” has been used commercially but was later reported to have low and inconsistent efficacy (Liu et al., 2013; Spadaro and Droby, 2016).

Aqueous “Natamycin” treatments followed by fruit coating reduced the incidence of decay from *P. digitatum* to <10% in grapefruit and lemons compared to the Natamycin untreated control at 81.9%. Moreover, experimental and commercial packing line studies have demonstrated that “Natamycin” mixed with fludioxonil or propiconazole applied as a storage fruit coating or aqueous flooder treatment typically resulted in >85% reduction of *P. digitatum* (Chen et al., 2020e). El Guilli et al. (2020) observed the effects of a *Pichia guilliermondii* strain Z1 granular-formulated product on “Valencia-late” oranges with a suspension of 10^5^ conidia/ml of *P. digitatum* and *P. italicum* at temperatures 4 and 20°C. Results showed the control achieved with strain Z1 on “Valencia-late” oranges was comparable to that of thiabendazole, indicating the promise of *Pichia guilliermondii* strain Z1 as a biofungicide against *P. digitatum* and *P. italicum*. Moreover, the strain Z1 is compatible with various waxes used in citrus packinghouses (Lahlali et al., 2014).

The major drawback in the commercialization of bioproducts based on biological control agents is the failure to provide consistent and reliable disease control in field experiments. Despite the growing demand for bioproducts, this failure hinders adoption and has slowed dissemination (Spadaro and Droby, 2016; Abbey et al., 2019). While biocontrol products alone do not provide complete control over green and blue molds, enhancements with mixtures of beneficial organisms, integration with low doses of fungicides, and adjustment of storage atmosphere can increase their protective powers (Spotts et al., 2002).

## Chitosan-Based Citrus Coatings

One of the most abundant polysaccharides in nature, chitosan is a biodegradable biopolymer obtained from deacetylation of the chitin present in the exoskeleton of crustaceans (e.g., crabs, lobster, shrimp; Hafdani and Sadeghinia, 2011; Talibi et al., 2014; Zhang et al., 2014). It exhibits direct antimicrobial properties against various microorganisms, including fungi, yeasts, and bacteria, and specifically against the post-harvest diseases *P. digitatum* and *P. italicum* in citrus (Palou et al., 2015). Citrus fruits in packing lines are often coated with wax-based compounds amended with conventional fungicides such as imazalil or thiabendazole to reduce weight loss, improve appearance, and control the post-harvest disease. Chitosan and its derivatives are the most-tested antifungal edible coatings to replace commercial waxes against *P. digitatum* and *P. italicum* formulated either alone or with other antifungal ingredients (Palou et al., 2015). The progress of edible antifungal coatings as a safe technology can address post-harvest physiological and pathological problems in citrus.

Past studies have investigated the effectiveness of chitosan to control green mold and blue mold diseases. Panebianco et al. (2014) conducted *in vitro* and *in vivo* assays with chitosan applications at 0.02–0.5% concentration levels against *P. digitatum*. *In vitro* tests showed that concentrations higher than 0.1% completely inhibited the pathogen growth. Moreover, *in vivo* assays demonstrated chitosan application at a concentration of 0.5% significantly reduced green mold on “Washington Navel”, “Valencia” oranges, “Femminello” lemons, and “Marsh Seedless” grapefruits (Panebianco et al., 2014). In another study, El Guilli et al. (2016) investigated the effects of chitosan on green mold and citrus fruit quality by wounding treated fruits with different concentrations of chitosan 24 h before inoculation with *P. digitatum*. Both *in vitro* and *in vivo* results revealed improved antifungal activity against *P. digitatum* with increased chitosan concentrations as well as enhanced chitinase and glucanase activities and elicitation of biochemical defense responses without impairing fruit quality. Chitosan not only reduced disease incidence and severity but also enhanced the activities of several enzymes such as SOD, peroxidase, hydrogen peroxide (H_2_O_2_), and the levels of glutathione in “Navel” oranges inoculated with *P. digitatum* and *P. italicum* (Zeng et al., 2010).

Researchers have explored the antifungal activity of chitosan coatings in combination with other antifungal treatments such as plant extracts, biological control antagonists, salts, and essential oils before or after the application of chitosan-based coatings. El-Mohamedy et al. (2015) observed the effects of coatings amended with chitosan and essential oil on citrus fruits as fungicide alternatives to control post-harvest diseases. A combination of chitosan, lemongrass essential oil, citral essential oil, and chitosan-essential oil mixtures significantly reduced the growth and spore germination of *P. digitatum* and *P. italicum in vitro* (El-Mohamedy et al., 2015). Similarly, Shao et al. (2015) observed that chitosan combined with clove oil inhibited *P. digitatum* mycelial growth in Satsuma mandarins *in vivo* and exhibited high antifungal activity *in vitro* with stimulation of fruit defense enzymes. Tayel et al. (2016) evaluated fungal chitosan from *Mucor rouxii* and plant extracts from cress seeds, pomegranate peels, olive leaves, and senna pods. *In vitro* qualitative and quantitative assays found that all of these agents exhibited antifungal activity against *P. digitatum* and *P. italicum*, resulting in inhibition of fungal growth and viability; however, *P. digitatum* was more resistant than *P. italicum* towards the examined agents (Tayel et al., 2016). In general, chitosan-amended coatings gradually release preservatives and provide additional properties for fruit quality maintenance and fungal growth inhibition (Galed et al., 2004). According to Cháfer et al. (2012), thymol, oil of thyme, has greater antifungal activity against *P. italicum* when incorporated with the chitosan coating on “Powell Navel” oranges without affecting fruit quality attributes.

In recent work, the antifungal activities of carboxymethyl chitosan (CMCS) combined with *Cryptococcus laurentii* controlled the spore germination of *P. italicum* in grapefruit. Combined treatments of CMCS and *C. laurentii* exerted a significant synergistic effect resulting in smaller lesion diameter, reduced decay incidence, and stimulation of defense enzyme activities with no impairment of fruit quality parameters (Wang et al., 2019). Likewise, Waewthongrak et al. (2015) evaluated the effect of *Bacillus subtilis* ABS-S14 endospores, a crude extract from its culture medium, cyclic lipopeptide antibiotics, and chitosan on the suppression of *P. digitatum* in mandarin fruit. Efficacy tests showed a significant reduction of fruit decay and induction of defense-related enzymes such as peroxidase and phenylalanine ammonia-lyase in the infected flavedo tissues. Furthermore, the combination of *Candida saitoana* with glycolchitosan was more effective in reducing *P. digitatum* infection in lemons and oranges as compared to stand-alone treatments matching the fungicide imazalil in effectiveness (El-Ghaouth et al., 2000). Pretreatment with sodium carbonate salts followed with a combination of *C. saitoana* and glycolchitosan most effectively controlled green mold in light green and yellow lemons (El-Ghaouth et al., 2000).

Research into the combined effects of salicylic acid (SA) and chitosan on the control of *P. digitatum* in grapefruits showed significantly reduced lesion diameter and disease incidence in comparison to applications of chitosan or SA individually. It also enhanced the β-1,3-glucanase, chitinase, PAL, peroxidase, and PPO activities and stimulated the synthesis of total phenolic-compound content without impairing post-harvest quality (Shi et al., 2018). Besides the use of chitosan and its derivative, several other edible antifungal coatings applied alone or in combination with other treatments were effective against citrus green and blue mold. For instance, Velásquez et al. (2014) observed the effect of pectin-based edible coatings made with essential oil against *Penicillium* sp. on “Valenica” oranges and found a concentration 1.5% essential oil reduced decay by 83% with improved shelf life. Essential oils from *Mentha spicata* and *Lippia scaberrima* integrated into commercial citrus waxes also stemmed in protective activity against green mold on “Valencia” and “Tomango” oranges (Du Plooy et al., 2009). Wax coatings not only reduce weight loss in oranges but also maintain overall fruit quality. Formulation of wax coatings with the essential oils carvacrol and thymol reduce green mold incidence and ethylene production in lemons artificially inoculated with *P. digitatum* (Pérez-Alfonso et al., 2012). Moreover, citral incorporated into commercial carnauba wax and applied to citrus fruit showed a concentration 10 times the minimum fungicidal concentration was required to decrease the incidence of *P. digitatum* (Fan et al., 2014). Valencia-Chamorro et al. (2008) developed and optimized hydroxypropyl methylcellulose -lipid edible composite films formulated with food additives or GRAS salts to inhibit the *in vitro* growth of *P. digitatum* and *P. italicum*. Subsequently, the authors also tested the curative activity of selected coatings *in vivo* on oranges and mandarins and found that coatings containing the GRAS salts potassium sorbate, sodium benzoate, sodium propionate, and their mixtures most effectively reduced green and blue mold (Valencia-Chamorro et al., 2009).

The quality maintenance and extended shelf life of chitosan-coated fruits suggest that chitosan application can partly substitute for synthetic fungicides in commercial storage and marketing (El Guilli et al., 2016). Limited studies exist on combining chitosan with GRAS salts, microbial antagonists, and physical treatment methods to enhance control of citrus decay, and further research is warranted.

## Synthetic Elicitors

Nowadays, industry favors synthetic chemicals that induce plant immunity and natural disease resistance to activate, bolster, or prime plant defense machinery over biocidal agrochemicals (Droby et al., 2002; Liu et al., 2010; Nantawanit et al., 2010; Zhou and Wang, 2018). The induction of natural resistance to pathogens in harvested fruit using non-toxic chemical elicitors in place of chemical fungicides is a promising and ecologically friendly approach for controlling post-harvest diseases (Zhou and Wang, 2018). Advantages to inducing disease resistance with elicitors include the ability to fight pathogens, lower costs than specific biological antagonists, environmental safety and friendliness, and effectiveness at all stages of fruit development, including preharvest and post-harvest (van Hulten et al., 2006; Conrath et al., 2015).

The SA has antifungal properties against specific pathogens in citrus, mango, and pear (Joyce et al., 2001; Shaat and Galal, 2004; Cao et al., 2006). Increasing SA concentrations through the exogenous application or endogenous synthesis stimulates systemic acquired resistance in plants (Verberne et al., 2000). It also delays senescence, retards fruit decay, and facilitates plant growth regulation and interaction with other organisms in response to biotic or abiotic stresses (Yalpani et al., 1994; Senaratna et al., 2000).

The plant hormone MeJA, formed *via* the octadecanoid pathway together with jasmonic acid (JA) (Holopainen et al., 2009), has been found to mediate diverse developmental processes and defense responses (Cheong and Do Choi, 2003) and enhance the disease resistance (Meng et al., 2009). Similarly, JA is a natural inducer of disease resistance that stimulates antifungal activity in crops such as mango, pear, and citrus fruits and regulates plant growth and development (Shaat and Galal, 2004; Yao and Tian, 2005).

The chemical elicitor β-aminobutyric acid (BABA) is a non-proteinogenic amino acid that behaves as a safe priming molecule of systemic resistance induction in several crops such as apple, citrus, and strawberry (Conrath et al., 2015; Baccelli et al., 2017; Guolin et al., 2019; Aghdam et al., 2020). In plants, BABA acts on various post-harvest fungi (Wang J. et al., 2018; Cheng et al., 2019) with multiple biochemical and physical defense mechanisms, including the creation of physical barriers (callose, lignin, and papillae), hypersensitivity reaction, accumulation of phytoalexins, induction of pathogenesis-related (PR) proteins, biosynthesis of terpenoids, generation of reactive oxygen species (ROS) with H_2_O_2_ and activation of defense pathways mediated by abscisic acid, SA, and JA (Cohen, 2002; Walters et al., 2013).

Iqbal et al. (2012) studied the effect of two organic elicitors, SA and MeJA, in “Lane Late” sweet orange, including pre- and post-harvest application for resistance induction. *In vitro* experiments with post-harvest treatment showed that SA ≥6 mM substantially inhibited the sporulation, radial growth, and spore germination of *Penicillium* sp. compared to MeJA and control. However, MeJA showed only a suppressive effect on fungal propagules at concentrations ≥4 mM. In contrast, preharvest spray application of 8 mM SA and 3 mM MeJA to “Lane Late” orange effectively reduced wound rotting, colony/lesion diameter, and spore mass density of *P. digitatum*. Overall, pre- and post-harvest treatments of fruit with SA proved more effective in lessening mold severity than MeJA.

Similarly, Shaat and Galal (2004) assessed the preharvest spray application of SA on the incidence of *P. digitatum* in grapefruit, lime, mandarin, and six orange cultivars. In general, preharvest application of elicitors proved more effective as it allowed the host to develop more induced resistance and fruit protection than in post-harvest application. *In vitro* growth inhibition of *P. digitatum* was highest with 400 mg L^−1^ SA treatment. A recent study examined the capacity of SA and JA to suppresses *P. digitatum* and *P. italicum* in post-harvest infection on *Citrus reticulata* “Kinnow,” *Citrus limon* “Meyer Lemon,” and *Citrus limetta* “Mosambi.” SA and JA significantly reduced the severity of *P. digitatum* and *P. italicum* on all tested citrus species compared to the non-treated control. The efficacy of both SA and JA in reducing disease severity depended on concentration; higher concentrations resulted in a greater degree of suppression. Results also showed that SA and JA increase the activity of PPO and peroxidase, which suppressed the development of green and blue mold most effectively in *C. reticulata* and least so in *C. limon* (Moosa et al., 2019).

In addition, INA (2,6-dichloroisonicotinic acid), a synthetic analog of SA, was investigated for controlling the post-harvest incidence of *P. digitatum* and *P. italicum*. Treatments of 1.0 mmol L^−1^ INA significantly reduced green and blue molds on both wound-inoculated and naturally infected fruit compared with the control. Moreover, β-1,3-glucanase, chitinase, PAL, peroxidase, and PPO can be used to control diseases (Jing et al., 2020).

Elsherbiny et al. (2021) studied the mechanisms and effects of BABA treatment on the inhibition of *P. digitatum* both in orange fruit and *in vitro*. BABA at 125 mM was found to significantly inhibit spore germination, mycelial growth, and germ tube elongation of *P. digitatum* and suppress disease incidence and disease severity compared to untreated fruit. In accordance with this study, Porat et al. (2003) found that treatments with BABA at 20 mM reduced the incidence of *P. digitatum* in infected wounds on grapefruit. Panebianco et al. (2014) likewise reported that very high concentrations of BABA reduce *P. digitatum* decay on oranges cvs. “Tarocco” and “Valencia” and on grapefruit cv. “Marsh Seedless” by 70–90%. The fungistatic properties of BABA affect the fungal cell membrane by inhibiting *P. digitatum* growth, increasing cell membrane permeability and malondialdehyde content, and decreasing the ergosterol and the total lipid contents. Similar structural defects have been observed in *P. digitatum* treated with cecropin A-melittin hybrid peptide BP21 (Wang W. et al., 2018), the essential oil of *C. reticulata* (Tao et al., 2014), and pinocembroside isolated from *Ficus hirta* Vahl. fruit (Chen et al., 2020d).

As biological control does function across a spectrum as broad as chemical fungicides, combining biocontrol agents with synthetic elicitors can enhance their performance. Zhou et al. (2014) investigated the effects of *Pichia membranaefaciens* and SA for the control of *P. digitatum* and *P. italicum* in citrus fruit. Combining the yeast with SA effectively enhanced the phenylalanine ammonialyase, peroxidase, polyphenoloxidase, chitinase, and β-1,3-glucanase activities and synthesis of phenolic compounds without ruining fruit quality parameters. In line with Zhang et al. (2010), these results suggested that SA enhanced the biocontrol efficacy of this yeast in post-harvest diseases by facilitating its growth and promoting nutrient and space competition.

Similarly, Guo et al. (2014) examined the preventive activity of MeJA alone and in combination with the antagonistic yeast *C. laurentii* for preventing green mold in citrus fruit. MeJA alone with a concentration of 100 μmol/L inhibited disease incidence and lesion diameter of mold decay compared with the control or the application of 100 μmol/L combined with *C. laurentii* at 1 × 10^8^ cells/mL. Relative to single-treatment groups and the control, MeJA and *C. laurentii* induced higher peroxidase, polyphenol oxidase, and catalase activity and a rise in the mRNA expression of PR5 (pathogenesis-related protein family 5); it likewise induced natural resistance and stimulated the proliferation of antagonistic yeast on the fruit surface. Additionally, when Zhou et al. (2018) applied SA (2.5 mmol L^−1^), *P. membranaefaciens* (1 × 10^8^ cells mL^−1^), or oligochitosan (15 g L^−1^), these exogenous elicitors led to effective inhibition of *P. digitatum* and *P. italicum* in pathogen-inoculated citrus fruit. Results indicated that the activation of the phenylpropanoid biosynthesis pathway led to the induction of resistance in citrus fruit.

These studies suggest potential strategies by which combinations of two to three different methods complement each other to suppress post-harvest disease in citrus; however, the results do not fully predict control efficacy under commercial practice. Therefore, additional research under commercial practice is warranted. Because little is known about the combined effects of elicitors on the metabolic pathway and regulatory network of phenylpropanoids in citrus fruit, further study into the interaction of citrus species with *Penicillium* sp. is needed to understand how different hosts respond to various treatments during storage and commercial application (Louw and Korsten, 2015).

## Food Additives

Food additives, particularly preservatives or substances classified as GRAS by the U.S. Food and Drug Administration are alternatives to conventional fungicides for post-harvest disease control in citrus fruits. The food industry commonly adds organic and inorganic salts to food for the purposes of leavening, pH control, taste, and texture modification (Smilanick et al., 1999). These compounds also exhibit a broad spectrum of activity against bacteria and fungi. The inorganic salts most widely used to control post-harvest disease in citrus fruit are sodium carbonate, sodium bicarbonate, and potassium sorbate, all classified as GRAS compounds (Table 3). Several other food-grade preservatives that have proven effective against citrus green and blue mold diseases include sodium paraben salts, sodium benzoate, and potassium silicate (Montesinos and Palou, 2016). Major advantages of using these salts for post-harvest treatment include antimicrobial properties, low toxicity, relatively low cost, ready availability, safety for humans and the environment, and its unrestricted use (El-Mougy et al., 2008; Deliopoulos et al., 2010; Palou, 2016).

**Table 3:** Salts and food additives for the control of *P. digitatum* and *P. italicum*.

**Pathogens**	**Salts**	**Fruits**	**References**
*P. digitatum*	Acetic acid, formic acid, and propionic acid	Grapefruit and oranges	Sholberg, 1998
	Sodium propionate	“Valencia” oranges	Hall, 1988
	Calcium chloride	Grapefruit	Droby et al., 1997
	Calcium polysulfide	Oranges and lemons	Smilanick and Sorenson, 2001
	Potassium sorbate	“Valencia” oranges	Smilanick et al., 2008
*P. italicum*	Sodium salicylate, sodium sulfite, boric acid, copper sulfate, and sodium ethylenediaminetetraacetic acid	Mandarin (*Citrus reticulata* Blanco) cv. clementine	Askarne et al., 2013
	Ammonium carbonate	Oranges, lemons	Askarne et al., 2011; Montesinos-Herrero et al., 2011
	Sodium ethylparaben	“Valencia” oranges	Moscoso-Ramírez et al., 2013
	Sodium hydrosulfide	Mandarins and oranges	Fu et al., 2014
*P. digitatum* and *P. italicum*	Sodium carbonate Sodium bicarbonate	Oranges, mandarins, and lemons	Plaza et al., 2004b; Youssef et al., 2012b; Askarne et al., 2013
	Sodium benzoate	“Valencia,” “Lane Late” oranges, lemons, and “Ortanique” mandarins	Montesinos-Herrero and Palou, 2016; Montesinos-Herrero et al., 2016
	Benzoic acid	Lemons	El-Mougy et al., 2008
	Potassium sorbate	“Valencia” oranges	Smilanick et al., 2008; D'Aquino et al., 2013
	Sodium metabisulfite and potassium metabisulfite	Oranges	Martínez-Blay et al., 2020
	Sodium dehydroacetate	“Ponkan” tangerines (*Citrus reticulata* Blanco)	Duan et al., 2016
	Calcium chelate Sodium silicate Potassium carbonate	“Comune” clementine and “Valencia late” oranges	Youssef et al., 2012b Palou et al., 2001

*In vivo* and *in vitro* studies have demonstrated the high effectiveness of salts such as potassium sorbate and sodium benzoate against *P. digitatum* and *P. italicum* decay in oranges and lemons (Palou et al., 2002a; Montesinos-Herrero et al., 2016). Non-toxic and tasteless sodium benzoate is specifically known for its bactericidal and bacteriostatic properties (El-Mougy et al., 2008). Likewise, *in-vivo* assays with sodium carbonate, ammonium carbonate, boric acid, copper sulfate, sodium ethylenediaminetetraacetic acid, sodium salicylate, sodium sulfite, and sodium metabisulfite are known to inhibit mycelial growth of *P. italicum* in citrus (Askarne et al., 2013). These salts act against molds through membrane disruption, stresses on pH homeostasis through anion accumulation within the cell, inhibition of essential metabolic functions, and activation of defense mechanisms in fruits (Smilanick et al., 2005; Youssef et al., 2014). Although these salts provide good control of citrus molds, application time is crucial because salts applied before the harvest period have more time to interact with mold pathogens than after harvest, resulting in greater efficacy (Youssef et al., 2012b).

Post-harvest *P. digitatum* and *P. italicum* incidence on lemons and oranges was effectively controlled by fumigation with ammonia gas not exceeding 6,000 μL/L (Montesinos-Herrero et al., 2011). Oranges treated with ammonium molybdate and sodium molybdate have shown a significant decrease in the incidence of *P. digitatum* and *P. italicum* (Palou et al., 2002a). Ammonium molybdate can inhibit acid phosphatase, which interferes with phosphorylation and dephosphorylation and affects metabolic processes in several organisms (Mukhopadhyay et al., 1988; Bodart et al., 1999). Fumigation of mandarins and oranges by hydrogen sulfide salts decreases the growth of *P. italicum* on tested fruit surfaces, inhibiting spore germination through ROS related mechanisms (Fu et al., 2014). Moreover, food preservatives are as successful as salts in controlling citrus molds. Sodium dehydroacetate, a common food preservative, inhibited mycelial growths of *P. digitatum* and *P. italicum in vivo* and *in vitro* experiments (Duan et al., 2016).

Several salts are known to induce host resistance *via* stimulation of defense-related genes. Electrolyzed sodium bicarbonate induced oxidative stress in the *P. digitatum* conidia *via* accumulation of ROS, the collapse of mitochondrial membrane, disrupted adenosine triphosphate production, and upregulated defense-related gene coding for peroxidase and PAL (Fallanaj et al., 2016). Furthermore, the electrolytes sodium metabisulfite, potassium sorbate, potassium carbonate, and sodium chloride were used to generate alkaline- (alEW), and acidic- (acEW) electrolyzed water for inhibition of *P. digitatum* and *P. italicum* on “Valencia” sweet orange (Youssef and Hussien, 2020).

Several studies conducted to control *Penicillium* molds suggested the performance of these salts could be enhanced by combining them with other treatments such as antagonistic microorganisms, hot water, low-dose chemical fungicides, and wax coatings (Smilanick et al., 2008; Youssef et al., 2012a). Teixidó et al. (2001) evaluated the potential of *Pantoea agglomerans* (strain CPA-2) in combination with sodium carbonate or bicarbonate solutions under ambient (20°C) and cold storage (3°C) conditions for the control of *P. digitatum* and *P. italicum*. The study detected a 97.6% reduction of decay incidence with sodium bicarbonate. In addition, Smilanick et al. (1999) observed a significant improvement in the effectiveness of sodium bicarbonate and carbonate when treatments were followed with *Pseudomonas syringae* ESC-10. The residues of biological control antagonists were found to persist long after treatment, thereby protecting fruit from reinfection.

Moreover, the combined application of marine yeast *Rhodosporidium paludigenum* and sodium bicarbonate proved as effective as a fungicide, eliminating the decay incidence of green mold in citrus fruit (Zhu et al., 2013). Also, Lu et al. (2018) assessed the effectiveness of the combined treatment of ammonium molybdate and *R. paludigenum* to control green mold disease in satsuma mandarin. The addition of 0.1 mmol L^−1^ ammonium molybdate significantly enhanced the biological activity of *R. paludigenum* against *P. digitatum*, reduced disease incidence by 89.3%, and discontinued mold development within 0–12 h of infection. Ammonium molybdate depresses the ecto-phosphate activity of *P. digitatum*, disturbs the environmental acidification of the pathogen, and suppresses spore germination.

Sorbic acid salts (also used as food additives) such as potassium sorbate are classified as minimal-risk active ingredients, similar to chemical fungicides in effectiveness, and appropriate for aqueous application (Smilanick et al., 2008). Heated aqueous solutions were found to enhance the performance of potassium sorbate against green mold. Inhibitory effects of sorbic acid on *P. digitatum* and *P. italicum* include inhibition of enzymes and protein synthesis, alteration of cell-membrane and cell-transport function, and uncoupling of oxidative phosphorylation in mitochondria (El-Mougy et al., 2008). Also, short dip treatments of citrus fruits in salt solutions of 2–3% have significantly reduced the incidence of *P. digitatum* and *P. italicum* without causing rind phytotoxicities (Palou, 2016). For instance, dips in sodium metabisulfite and potassium metabisulfite at 20 and 50 mM for 60 or 120 s at room temperature (20°C) significantly reduced the incidence and severity of *P. digitatum* and *P. italicum* on “Valencia” oranges (Martínez-Blay et al., 2020). Dip treatments of 60 s with 3% sodium benzoate heated above 50°C resulted in 90% reduction of *P. digitatum* and *P. italicum* on “Valencia” oranges, “Lanelate” oranges, “Fino” lemons, and “Ortanique” mandarins, offering an important disease control alternative for the commercialization of citrus without fungicidal residues (Montesinos-Herrero et al., 2016).

In addition, Cerioni et al. (2013) investigated the use of potassium sorbate, sodium bicarbonate, and potassium phosphite in combination with heat and H_2_O_2_ in the presence of copper sulfate to control *Penicillium* molds in lemons. The authors reported phosphite solutions controlled *P. digitatum* only when heated or combined with fungicides. Moreover, combining wax with potassium sorbate salts provided antifungal activity against citrus molds but impaired the film-forming capacity of the wax, eventually resulting in fruit weight loss (Youssef et al., 2012a; Parra et al., 2014). In a study by Youssef et al. (2012a), ammonium bicarbonate was found not to interfere with the capacity of wax to retard weight loss.

Although these treatments offer alternatives to fungicides for post-harvest disease control, concerns regarding dietary safety, disposal, fruit quality parameters, worker safety, and other regulatory issues must be addressed before these compounds gain approval for post-harvest use. For instance, the disposal of sodium bicarbonate raises regulatory issues in some locations because of its high pH, electrical conductivity, and sodium content (Smilanick et al., 2008). Further research from an integrated approach should seek a suitable combination of salts with non-chemical treatments such as wax to improve fruit quality parameters.

## Physical Control Methods

Physical treatments are gaining popularity in the control of citrus post-harvest diseases as it leaves no residue and has the least environmental impact than other options (Palou, 2009; Usall et al., 2016). Of the physical technologies so far investigated for reducing *P. digitatum* and *P. italicum* in citrus fruit and prolonging its storage life, the most promising include heat, ultraviolet light (UV-C and UV-B), blue light, x-rays, and gamma irradiation; complementary methods include controlled and modified atmospheres and cold storage (Droby et al., 1993; Nafussi et al., 2001; Kader, 2002; Smilanick et al., 2003; Palou et al., 2007; Gündüz and Pazir, 2013; Lafuente and Alférez, 2015; Jeong et al., 2016; Yamaga et al., 2016; Table 4).

**Table 4:** Physical methods for the control of *P. digitatum* and *P. italicum*.

**Treatments**	**Treatment intensity**	**Cultivars**	**Pathogens**	**References**
Hot water treatment	Dipping for 5 min at 50°C	Oranges and lemons	*P. digitatum*	Smoot and Melvin, 1963
	Brief immersions for 2–5 min at 45–55°C	*Citrus* spp.	*P. digitatum* and *P. italicum*	Spalding and Reeder, 1986; Couey, 1989; Rodov et al., 1995
	Dipping at 50–55°C for 150 s, incubated at 20°C for 7 days	Oranges and lemons	*P. digitatum* and *P. italicum*	Palou et al., 2001
	2–3 min hot water dipping at 50–53°C; 56°C for 20 s; Hot water rinse brushing (HWRB) at 56°C for 20 s; 63°C for 15 s; 62.8°C for 30 s	Oranges	*P. digitatum* and *P. italicum*	Schirra et al., 1997; Porat et al., 2000; Smilanick et al., 2003; Strano et al., 2014
	3 min hot water dipping at 53°C	Pummelo grapefruit hybrid “Oroblanco”	*P. digitatum* and *P. italicum*	Rodov et al., 1995
	HWRB at 56°C for 20 s	Tangerines, oranges, and red grapefruits	*P. digitatum*	Porat et al., 2000
	Hot water dipping at 52–53°C for 2 min. HWRB at 63°C for 15 s	Lemons	*P. digitatum*	Nafussi et al., 2001; Smilanick et al., 2003
	HWRB at 62°C for 20 s	Oranges and lemons	*P. digitatum*	Lanza et al., 2002
	HWRB at 56°C for 20 s	Tangerine, oranges, and red grapefruits	*P. digitatum*	Porat et al., 2000
	Hot water treatment at 56–60°C for 10 s	Pummelo grapefruit hybrid “Oroblanco”	*P. digitatum* and *P. italicum*	Rodov et al., 2000
	HWRB at 55°C for 20 s	Kumquats	*P. digitatum* and *P. italicum*	Ben-Yehoshua and Porat, 2005
	3 min hot water dipping at 56°C	*Citrus reticulata* Blanco × *Citrus sinensis* (L.) Osbeck	*P. digitatum*	Kyriacou, 2011
Curing	35°C for 72 h	Washington Navel oranges	*P. digitatum* and *P. italicum*	Tuset, 1987
	33°C for 65 h	Oranges and lemons	*P. digitatum* and *P. italicum*	Plaza et al., 2003, 2004a
	32°C in a saturated water atmosphere for 48 h	Lemons	*P. digitatum*	Stange and Eckert, 1994
	Two cycles intermittent curing for 18 h at 38°C	Mandarins	*P. italicum*	Pérez et al., 2005
	Curing at 40°C for 18 h	Oranges	*P. digitatum* and *P. italicum*	Nunes et al., 2007
	32°C for 3 days	“Femminello” lemons and “Valencia” oranges	*P. digitatum*	Lanza and Di Martino Aleppo, 1996
	36°C and longer exposure	Tarocco oranges	*P. digitatum*	Lanza and Di Martino Aleppo, 1996
	“Valencia,” “Pineapple” oranges, and “Flame” grapefruit at 30–35°C for 24 h or longer optimum condition for “Valencia” oranges and “Flame” grapefruit at 35°C with 95–100% humidity for 48 h	“Valencia” oranges, “Flame” grapefruit, and “Pineapple” oranges	*P. digitatum*	Zhang and Swingle, 2005
	30°C with high humidity (90–95%) for 72 h	Satsuma mandarins	*P. digitatum* and *P. italicum*	Kinay et al., 2005
Non-ionizing irradiation and UV-C	Fruit exposed to low doses of UV-C irradiation	Marsh seedless grapefruit	*P. digitatum*	Droby et al., 1993
	UV-C application at doses of 0.5 KJ m^−2^	Star Ruby grapefruit	*P. digitatum*	D'Hallewin et al., 2000
	Low UV-C irradiation (7.92 kJ m^−2^) inactivates spores on the surface of the fruit	Oranges	*P. digitatum* and *P. italicum*	Gündüz and Pazir, 2013
	The antifungal activity of lemon peel extracts was improved by short-time UV-B irradiation	Lemons	*P. digitatum*	Ruiz et al., 2017
	210 and 630 μmol m^−2^ s^−1^ LED blue light quantum fluxes of boosted scoparone in the flavedo	Oranges	*P. digitatum*	Ballester and Lafuente, 2017
	Low-intensity LED blue light irradiation lowered blue mold symptom development and suppressed fungal sporulation in satsuma mandarins	Mandarins	*P. italicum*	Yamaga et al., 2015
Ionizing irradiation	510 and 875 Gy X-ray irradiations decreased the sporulation of *P. digitatum* and *P. italicum* on mandarins that were treated with sodium carbonate	Mandarins	*P. digitatum* and *P. italicum*	Palou et al., 2007
	1.0 kGy gamma-irradiation demonstrated germ tube elongation, total inhibition of spore germination, and mycelial growth of *P. digitatum*	Mandarins	*P. digitatum*	Jeong et al., 2016
Cold storage	Temperatures of 3–5°C and relative humidity of 90–95%	Oranges and mandarins	*P. digitatum* and *P. italicum*	Kader, 2002
	Temperatures of 10–14°C	Lemons, limes, and grapefruit	*P. digitatum* and *P. italicum*	Kader, 2002
	Temperatures of 0–3°C	*Citrus* spp	*P. digitatum* and *P. italicum*	Tuset, 1987; Snowdon, 1990; Brown and Eckert, 2000a,b
Storage in a controlled atmosphere	5–10% O_2_ + 0–5% CO_2_	Oranges and mandarins	*P. digitatum* and *P. italicum*	Kader, 2002
	5–10% O_2_ + 0–10% CO_2_	Lemons, limes, and grapefruit	*P. digitatum* and *P. italicum*	Kader, 2002
Storage in ozonated atmosphere	200 μl/L ozone gas in humid air with 95% relative humidity at 5°C for 1 h	*Citrus* spp	*P. digitatum* and *P. italicum*	Margosan and Smilanick, 1998

Heat treatments can be applied to citrus fruit *via* hot water dips and sprays, hot vapor or curing (hot air application), and hot water rinsing and brushing. When infection structures are present on fruit surfaces, heat applied for a short period can easily affect these tissues, inducing several physiochemical changes to achieve a significant degree of control. Factors that determine the effectiveness of heat treatments include the product's condition prior to treatment, type of commodity, temperature, duration of treatment, and mode of heat application. Studies on the performance of water temperatures ranging from 40 to 65°C have been conducted (Palou et al., 2002a; García et al., 2016). Hot water treatment (HWT) was found to interrupt fungal spore growth for 24–48 h by the accumulation of secondary metabolites such as PR proteins, phytoalexins, accumulation of lignins in fruit infected by fungus, and production of ROS contributing to resistance against *P. digitatum* and *P. italicum* in citrus fruit (Nafussi et al., 2001; Yun et al., 2013; Perotti et al., 2015; Sui et al., 2016). Curing is another HWT post-harvest decay control method whereby citrus fruits are exposed for 2–3 days to air atmospheres heated to temperatures higher than 30°C at high relative humidity (RH >90%) (Palou, 2009). Improper heat treatments such as excessive temperatures and long durations have damaged fruit. For example, 53–55°C for 2–3 min. and 60°C for 20 s. have led to surface injury and rind browning in oranges (Schirra et al., 1997; Porat et al., 2000; Palou et al., 2001).

Both UV-C and UV-B are non-ionizing irradiations that have been widely studied for the prevention of green and blue mold in citrus. UV-B irradiation has been reported to harm the surface of citrus fruit less than UV-C treatment (Kaewsuksaeng et al., 2011) and at intensities higher than 30 kJ m^−2^ inactivated *P. digitatum* and *P. italicum* conidia *in vitro* (Yamaga et al., 2016). Factors determining the effectiveness of UV irradiation include type and intensity, harvesting period, stage of fruit development, and storage temperature (Droby et al., 1993; Yamaga et al., 2016). Overall, UV treatment causes metabolic and anatomical changes and accumulation in citrus flavedo of secondary metabolites such as polyphenols and phytoalexins, which are involved in fruit resistance (Droby et al., 1993; Ruiz et al., 2017). However, high intensities of UV-C irradiation might damage the flavedo of citrus and warrant precautions (Kim et al., 1991). Large-scale studies on UV treatment for citrus decay are needed to make any recommendations for the commercial use of irradiation in post-harvest citrus handling.

Blue light has the potential to reduce green and blue mold disease during post-harvest storage of citrus. It has been reported that blue light enhances fruit resistance against *P. digitatum* and *P. italicum* by stimulating the production of secondary metabolites and impairing fungal growth (Lafuente and Alférez, 2015; Ballester and Lafuente, 2017). For instance, Liao et al. (2013) found that blue light at a photon fluence rate of 40 μmol m^−2^ s^−1^ decreased the symptomatic development of green and blue mold in “Fallglo” tangerine and *in vitro* fungal growth of *P. italicum*, leading to the induction of defensive responses in the host. *In vitro* experiments also revealed that the efficacy of blue light increases with the duration of the application and light quantum flux by affecting fungal morphology and sporulation and increasing the phytoalexin scoparone and production of ROS in fungal cell walls (Lafuente and Alférez, 2015; El-Esawi et al., 2017).

Similarly, X-ray and gamma irradiation have been recognized as sustainable methods for extending the post-harvest life of citrus from decay (Rojas-Argudo et al., 2012; Guerreiro et al., 2016). X-ray differs from gamma irradiations in that X-rays are concentrated in the same direction as the electron beam while gamma rays are emitted uniformly in all directions (Palou, 2009). Rojas-Argudo et al. (2012) found that X-ray irradiation stimulates rind biosynthesis and synthesis of the phytoalexins scoparone and scopoletin in “Clemenules” mandarins at a storage temperature of 20°C. X-rays have increased scoparone levels after fruit inoculation in combination with sodium carbonate. The ionizing characteristics of X-ray irradiation was found to cause oxidative stress in fruits, which can influence the bioactive compounds located in fruit tissues and potentially improve the resistance of those fruits to pathogens (Oufedjikh et al., 2000).

Gamma radiation has likewise proven detrimental to fungal physiology by disrupting fungal cell membranes, retarding fruit ripening and respiration rate, and regulating the activity of enzymes (Cia et al., 2007; Schweiggert et al., 2007; Wang et al., 2017). However, Jeong et al. (2016) reported that higher doses may cause severe damage to the surface of citrus. Operating gamma-irradiation at lower doses can exclude this issue with other treatments such as sodium dichloro-s-triazinetrione (Jeong et al., 2016). Overall, if not applied correctly (in terms of dose, intensity, and duration), irradiation can adversely affect fruit quality parameters, stimulate phytotoxicities, and even impact human health. Combining irradiation treatment with other environmentally friendly techniques would help to minimize detrimental effects and increase efficacy.

Cold storage and storage in controlled or modified atmospheres are complementary tools that provide fungistatic activity by inhibiting or delaying the growth and development of pathogens. They also help reduce host metabolic activity, delay senescence, and maintain fruit resistance to fungal infection (Usall et al., 2016).

## Outlook and Prospects

Green and blue mold disease in citrus poses a major threat worldwide. The conventional disease management approaches involving synthetic fungicides are increasingly being questioned because of their potential deleterious impacts on human and environmental health, and the growing problem of fungi developing resistance to the synthetic fungicides. Non-toxic alternatives to synthetic fungicides include biological control, bio-fungicides, plant extracts and essential oils, chitosan, salts, hot water treatments and UV-radiation, and synthetic elicitors (Janisiewicz and Korsten, 2002; Droby et al., 2009). Although producers and consumers accept these new approaches, limited research has investigated the effectiveness of these methods on large-scale production. Not much is known about mechanisms of action of these alternative control approaches against green and blue mold disease, while the pathogen continues to cause huge economic losses. To manage these diseases, researchers might explore the molecular mechanisms of plant/fruit-pathogen interactions, including pathogenicity and plant resistance. Identification and functional analysis of citrus genes that regulate citrus fruit *via* transgenesis or genome editing may lead to the development of novel and durable control strategies against *P. digitatum* and *P. italicum*. Recent advances in the study of disease resistance in citrus are made possible, thanks to the availability of complete genome sequence of citrus, optimization of genetic transformation systems, and establishment of CRISPR/Cas9 gene-editing approaches. Thus, identification of resistant genes and developing resistant citrus varieties *via* genome editing might provide a promising pathway to control *P. digitatum* and *P. italicum* pathogens.

## Author Contributions

UB conceptualized the review, wrote the original draft, investigated, revised, and edited the draft manuscript.

## Conflict of Interest

The author declares that the research was conducted in the absence of any commercial or financial relationships that could be construed as a potential conflict of interest.

## Publisher's Note

All claims expressed in this article are solely those of the authors and do not necessarily represent those of their affiliated organizations, or those of the publisher, the editors and the reviewers. Any product that may be evaluated in this article, or claim that may be made by its manufacturer, is not guaranteed or endorsed by the publisher.
